# Beyond the Usual Suspects: Examining the Role of Understudied Histone Variants in Breast Cancer

**DOI:** 10.3390/ijms25126788

**Published:** 2024-06-20

**Authors:** Hejer Dhahri, Wesley N. Saintilnord, Darrell Chandler, Yvonne N. Fondufe-Mittendorf

**Affiliations:** 1 Department of Molecular and Cellular Biochemistry, University of Kentucky, Lexington, KY 40536, USA or hejer.dhahri@vai.org (H.D.); wsaintilnord@wustl.edu (W.N.S.); 2 Department of Epigenetics, Van Andel Research Institute, Grand Rapids, MI 49503, USA; darrell.chandler@vai.org; 3 Department of Genetics, Washington University School of Medicine, St. Louis, MO 63110, USA; 4 The Edison Family Center of Genome Sciences and Systems Biology, Washington University School of Medicine, St. Louis, MO 63110, USA

**Keywords:** histone variants, onco-histones, nucleosome stability, chromatin structure, gene regulation, breast cancer, histone chaperone, epigenetics, chromatin modifiers, PTMs

## Abstract

The incorporation of histone variants has structural ramifications on nucleosome dynamics and stability. Due to their unique sequences, histone variants can alter histone–histone or histone–DNA interactions, impacting the folding of DNA around the histone octamer and the overall higher-order structure of chromatin fibers. These structural modifications alter chromatin compaction and accessibility of DNA by transcription factors and other regulatory proteins to influence gene regulatory processes such as DNA damage and repair, as well as transcriptional activation or repression. Histone variants can also generate a unique interactome composed of histone chaperones and chromatin remodeling complexes. Any of these perturbations can contribute to cellular plasticity and the progression of human diseases. Here, we focus on a frequently overlooked group of histone variants lying within the four human histone gene clusters and their contribution to breast cancer.

## 1. Introduction

The regulated expression of histone genes and tissue-specific utilization of histone variants are intriguing aspects of chromatin structure that differ from the well-studied histone post-translational modifications (PTMs) [1,2,3]. Histone variants contribute to nucleosome structural diversity and chromatin architecture and regulate processes like DNA repair, centromere function, and gene activation or silencing [4,5,6,7,8,9]. However, our understanding of how histone variants are regulated, incorporated into chromatin, and interact with other regulatory proteins remains limited, especially in human diseases such as breast cancer.

Decades-long consensus has held that the development and progression of breast cancer is mediated by driver mutations in commonly known genes. These genetic alterations make individuals more prone to disease development and confer a competitive edge to the tumor in terms of growth. While genetic mutations have long been recognized as the key drivers of cancer development, emerging research has also highlighted the crucial role of epigenetic changes in this process. These modifications drive changes in gene expression that do not involve alterations in the underlying DNA sequence. The epigenetic aspect of the cell is defined by the status of DNA methylation, covalent modifications of histones, chromatin structure, and the network of chromatin modifiers. The integrity of this regulatory system is highly important to maintain normal gene expression regulation. In addition, like genetic mutations, aberrant changes to this epigenetic system can have profound effects on cellular behavior. Growing interest in this field has shown that dysregulation of these epigenetic events plays key roles in carcinogenesis and tumor progression. Several research studies have focused on the ‘histone code’ governed by the covalent modification of histones through methylation, acetylation, phosphorylation, ubiquitination, ADP-ribosylation, or SUMOylating and how this language translates into gene expression changes [10]. Beyond this, the incorporation of histone variants and onco-histones into the nucleosome is another mechanism used by the cell to regulate the structure of chromatin, histone modification, chromatin network, and ultimately the accessibility of regulatory elements to target gene promoters. In this paper, we review human histone isoforms and their epigenetic role(s) in breast cancer.

## 2. Canonical and Non-Canonical Histones

Eukaryotic cells wrap their nuclear DNA around histone proteins to form chromatin. The basic repeating unit of chromatin is the nucleosome, which consists of a histone octamer of four core histone proteins (two copies each of histone: H2A, H2B, H3, and H4). Nucleosome assembly involves the sequential deposition of (H3–H4)_2_ tetramer and H2A–H2B dimers onto DNA [11,12]. The nucleosome is stabilized via electrostatic interactions between the positively charged histones and the negatively charged DNA phosphate backbone [13]. Linker histone H1 then binds to the entry–exit nucleosomal DNA to form a chromatosome [14], to efficiently store and protect DNA from damage.

Histone deposition is tightly regulated by specific chaperones [15], especially during the S-phase of cell division, where timely and coordinated assembly of newly synthesized DNA into chromatin preserves genome integrity and regulates gene expression [16,17,18,19,20,21]. Cell division significantly increases the histone pool within a specialized subnuclear compartment called the histone locus body (HLB) [18,22], which facilitates spatial assembly of transcription factors and mRNA processing components around histone loci to promote rapid expression and processing of canonical histone mRNAs [23,24]. In eukaryotes, canonical histones lack introns and polyadenylated tails, and their mRNAs feature a unique 3′ end structure that is recognized by stem loop binding protein (SLBP) [20,25]. SLBP expression increases as cells enter the S-phase, where it helps stabilize interactions between U7 snRNP and histone downstream elements (HDEs), leading to endo-nucleolytic cleavage of canonical histone pre-mRNAs [26,27,28]. SLBP degradation at the end of the S-phase [21] results in decreased canonical histone mRNAs and proteins [21,29].

Although histones are among the longest-lived proteins, non-dividing cells outlive those histones that were initially deposited during cell division [9,30,31,32]. Thus, cells have evolved replication-independent mechanisms to incorporate non-canonical histones (i.e., histone variants) into chromatin outside of the S-phase. Numerous histone variants have been discovered in humans [33], differing in their primary sequence, introns, poly-A tails, and other mRNA characteristics [34,35,36]. These variants diverge from canonical histones by single amino acid changes or inclusion/deletion of entire protein domains, thus influencing chromatin architecture and gene regulation [2,37,38,39]. Histone variants and their specific chaperones recruit effector proteins and chromatin remodelers that also influence gene expression [15,40,41,42]. For example, H3.3 is deposited by HIRA and DAXX chaperones in actively transcribed regions during normal cell growth and development [43,44], changing nucleosome stability [45] and altering histone PTMs [46,47,48]. Nucleosomes containing H2A.Z enhance the accessibility of transcriptional machinery [49] to the promoters of estrogen receptor (ERα)-dependent genes in breast cancer [50]. Thus, histone variants shape chromatin structure and function in normal development [51,52] but are also involved in diseases like breast cancer [53,54].

## 3. Histone Gene Organization and Expression

**Figure 1:**
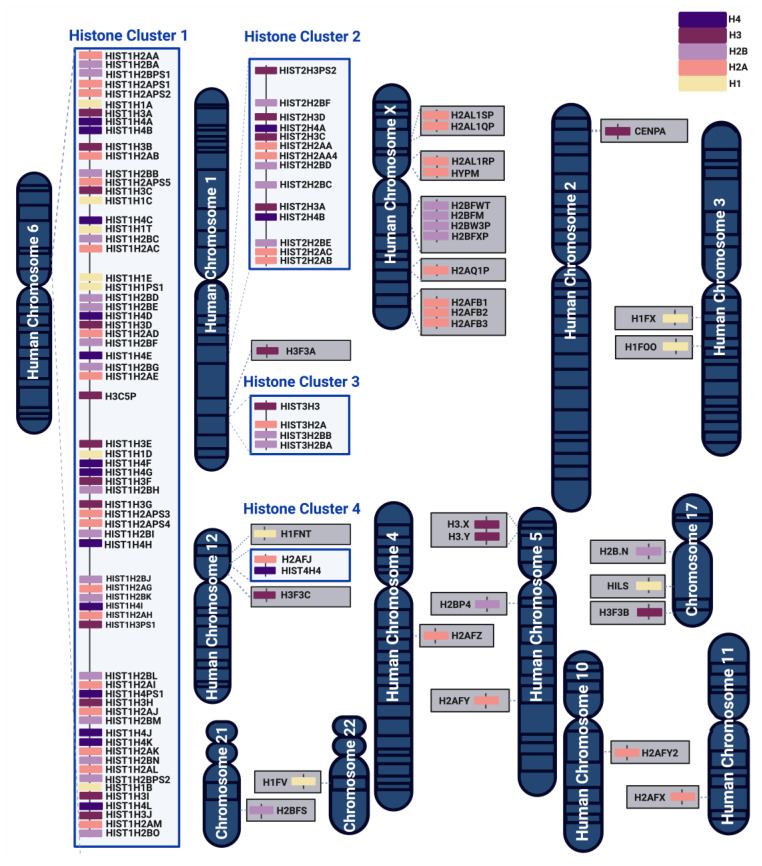
Chromosomal organization of human histone genes belonging to histone H4 family (in indigo), H3 family (in purple), H2B family (in lilac), H2A family (in salmon), and H1 family (in cream yellow). Most of the histone isoforms are organized in 4 histone clusters, whereas other histone genes are dispersed over various chromosomal regions. The arrangement and orientation of histone genes within the genome allow for their controlled expression by distinct promoters.

Human histone proteins (H1, H2A, H2B, H3, and H4) are encoded by a large family of genes, which can be clustered or dispersed across the genome (Figure 1 and Table A1). The major clusters include HIST1 on chromosome 6, HIST2 and HIST3 on chromosome 1, and HIST4 on chromosome 12. These clusters comprise canonical histones, histone variants, and pseudogenes, where some pseudogenes can also produce functional proteins [55,56]. In evolutionarily distant species like *Saccharomyces cerevisiae*, genes of partner histones (i.e., H2A and H2B that form dimers, and H3 and H4 that form tetramers) are arranged pairwise and are coordinately expressed from the same promoter region [18,57]. Initially, it was thought that clustered histone genes in eukaryotes are functionally identical and expressed at similar levels [58]. However, unlike in yeast, human histone genes and their interacting partners are not arranged in the same tandem repeat organization [4,59,60,61,62] (Figure 1). In fact, human histone genes are regulated by their own promoters and can be differentially expressed to carry out distinct spatial–temporal functions [59].

Histones within the major histone gene clusters are commonly believed to be expressed solely during cell division. Several recent studies, however, highlight findings that many of these histones are expressed outside of the S-phase and are independent of replication, for instance: 1. Certain histones within these clusters are expressed in non-dividing cells and are independent of SLBP [33,56,63]. As an example, recent studies show that several H2B variants within the human histone clusters are produced in non-dividing cells. These variants differ from canonical histone transcripts in that they possess a poly-adenylated tail and lack the stem loop structures [32,64,65,66,67,68,69,70]. 2. Genes involved in the expression and mRNA processing of histones were found to be expressed in terminally differentiated cells [65]. 3. Depletion of SLBP during the S-phase only modestly reduced the levels of histone mRNAs [71]. These studies therefore show that certain histones are expressed outside of the replication phase, hence the term ‘replication-independent’ histones [70]. Indeed, studies now show that histone genes within histone gene clusters are differentially expressed and are not only ‘replication-independent’, but their expression is cell-type- and tissue-type-specific [7,8,59,72,73,74]. For example, *HIST1H2AE* shows high expression in breast tissue, indicating its significance in breast tissue specificity [75]. The differential expression of histone variants across cell types suggests their diverse physiological roles [8,59], some of which are outlined below.

## 4. Histone Isoforms and Nucleosome Structure

### 4.1. Histone H2A Isoforms

**Figure 2:**
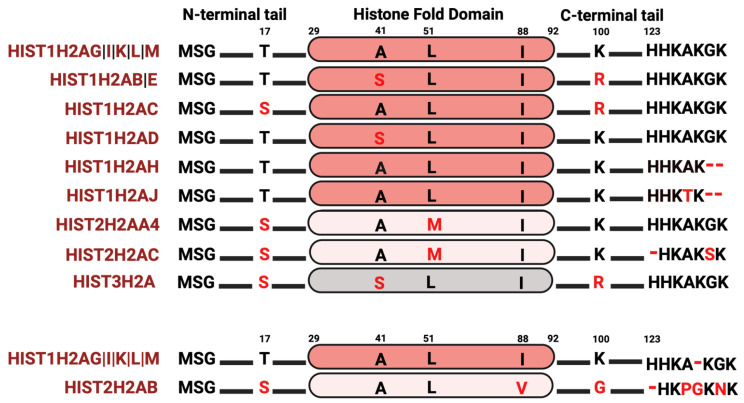
H2A isoform sequence alignments. The most abundant histone variant is indicated at the top. Red letters indicate divergent amino acids relative to the most abundant isoform.

Among the core histone proteins, histone H2A exhibits the highest number of variations. Histone H2A contains a C-terminal docking domain critical in linking the H3–H4 tetramer with the H2A–H2B dimer, with consequences in octamer stability. Additionally, the interaction between two H2A molecules in a nucleosome stabilizes the DNA helix at the back face of the nucleosome. H2A also makes a substantial contribution to the nucleosome surface, facilitating intra-nucleosomal interactions necessary for chromatin compaction; thus, changes in amino acid composition may have a profound effect on nucleosome stability, chromatin structure, and regulation of gene expression. Although several H2A variants, such as macroH2A, H2A.X, and H2A.Z, have been extensively studied [49,76,77,78], the extent of research into the histone H2A variants that are found within the clusters is still limited. Within the histone clusters, the most prevalent H2A protein, known as the “canonical” H2A protein, is encoded by five distinct genes (Figure 2). In analyzing the histone variants, a few of the H2A variants have a serine at position 17 instead of a threonine, compared to the “canonical” histone H2A protein. Phosphorylation at S17 is crucial for responding to DNA damage and hydrogen-peroxide-induced stress [79,80,81], hindering p53 binding protein 1 (53BP1) activity at damaged DNA sites and reducing neuronal apoptosis [82]. Given that several serine–threonine kinases have preferences either for serine or threonine as a phosphate acceptor, the S17T substitution can either add or delete a potential phosphorylation site [83,84], consequently impacting the nucleosome interactome. In addition, some of these H2A variants have amino acid changes in the histone fold domain, including A41S, L51M, and I88V. While some of these are typically considered as “conservative” substitutions in proteins, they can alter histone–histone interactions, PTMs, and ultimately gene expression patterns [85]. The H2A C-terminus exhibits the most sequence diversity, not only in amino acid substitutions but also in sequence length. Since the H2A C-terminus is positioned near the DNA entry and exit site, amino acid changes in this region could influence nucleosome breathing (the transient opening of ~10 bp of DNA from the nucleosome) and DNA accessibility by regulatory factors. Indeed, several studies have shown that the C-terminal tail of H2A is critical in the wrapping and unwrapping of nucleosomal DNA [49,86,87,88,89]. Apart from direct nucleosomal effects, some of these changes could impact PTMs on histones. Arginine residues can be methylated; therefore, the L100R substitution seen in several H2A variants may also be subjected to methylation with consequences in gene expression [8,90,91]. Thus, even subtle amino acid substitutions can alter the structure of a single nucleosome or introduce modifications (such as histone methylation) that influence gene expression.

### 4.2. Histone H2B Isoforms

**Figure 3:**
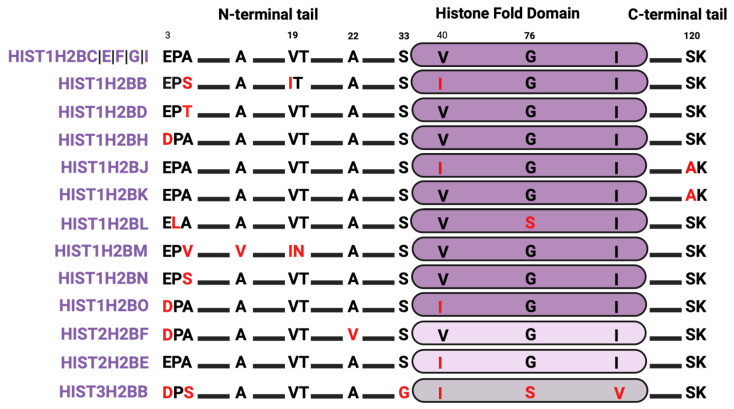
H2B isoform sequence alignments. Alignments are relative to the most abundant isoform. Red letters indicate divergent amino acids relative to the most abundant isoform.

Most of the sequence diversity in H2B variants is concentrated in the lysine-enriched N-terminal tails (Figure 3), potentially impacting nucleosome structure. For instance, the P4L substitution in HIST1H2BL changes an amino acid with a cyclic side chain to a residue with a free α-amino group, which should destabilize the structure. The A5S/T substitution in several H2B isoforms introduces a polar (-OH) side chain that could also impact nucleosome structure. Because S and T can be phosphorylated, a change from A to S/T at position 5 might also change the phosphorylation status at this site. Additionally, the A5S/T substitution neighbors a K6 that is frequently methylated and acetylated during the epithelial-to-mesenchymal transition in breast cancer, so changes to S/T might impact potential PTMs of this neighboring site [92]. For example, the residue H2B E35 is one instance of a neighboring effect, where H2B S36 phosphorylation is impaired by E35 ADP-ribosylation [93]. S33 is normally phosphorylated by the signaling kinases adenosine monophosphate (AMP)-activated kinase (AMPK) and ribosomal protein S6 kinase 1 (S6K1) to activate stress response genes and regulate early adipogenesis pathways [94,95], but S33 is replaced by glycine in the HIST3H2BB isoform. Finally, histone HIST1H2BJ and HIST1H2BK variants harbor an alanine instead of serine at position 120, which could alter hydrogen bonds and nucleosome integrity or delete a phosphorylation site.

### 4.3. Histone H3 Isoforms

**Figure 4:**
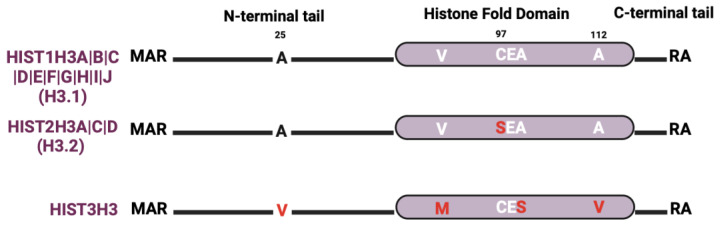
H3 isoform sequence alignments. Alignments are relative to the most abundant isoform. Red letters indicate divergent amino acids relative to the most abundant isoform.

The most studied histone H3 variant is H3.3, which is encoded by three genes (*H3F3A*, *H3F3B*, and *H3F3C*) that are distributed outside of the histone gene clusters (Figure 1). There are relatively few H3 protein isoforms within the histone gene clusters even though there are many H3 genes (Figure 4). The most abundant clustered histone H3 protein is HIST1H3A|B|C|D|E|F|G|H|I|J (also known as histone H3.1). HIST2H3A|C|D (also known as histone H3.2) differs from histone H3.1 by a single amino acid at position 97 (serine-to-cysteine substitution). While crystallography studies suggest that histone H3.1 and H3.2 have no discernable effect on nucleosome structure, S97 in the H3.2 variant is on the H3–H4 tetramer accessible surface, which makes it a possible interaction site for H3.2-specific chaperones [96]. Recent studies also show that H3.1 and H3.2 differ in their PTMs. For instance, H3.1 is enriched in K14 acetylation (a mark associated with gene activation), while H3.2 is enriched in K27 di- and tri-methylation (marks linked to gene silencing) [97,98]. Lastly, HIST3H3 contains four amino acid substitutions (A25V, V72M, V99A, and A112V) that make nucleosomes much more unstable than nucleosomes containing H3.1 or H3.2 by weakening the association of the HIST3H3–H4 tetramer with H2A–H2B dimers [34,99].

### 4.4. Histone H4 Isoforms

**Figure 5:**
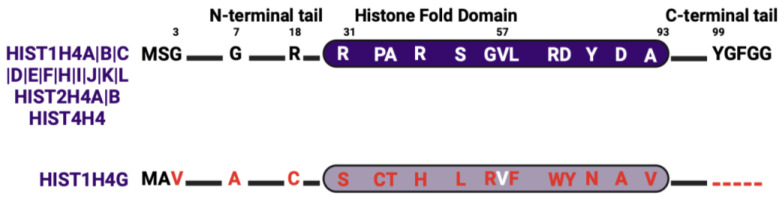
H4 isoform sequence alignment. Alignments are relative to the most abundant isoform. Red letters indicate divergent amino acids.

As of now, all identified histone H4 genes are distributed within the histone gene clusters (Figure 1), and all encode for the same amino acid sequence except for one isoform, HIST1H4G. The protein sequence of the HIST1H4G variant has substantial differences from the most abundant H4 protein throughout all domains (Figure 5). HIST1H4G differs from canonical H4 by 20 amino acid residues. These substitutions have significant implications for nucleosome stability and the regulation of histone PTMs [100]. In the N-terminal domain, the conformationally flexible glycine is replaced by the hydrophobic valine and alanine at positions 3 and 7, respectively. These substitutions in HIST1H4G increase its N-terminal tail hydrophobicity, which could impact histone tail bridging and the positioning of nucleosomes [101,102]. This is because H4 histone tails play a significant role in the attractive interaction between nucleosomes [103,104]. R18 (with its positively charged side chain) is replaced with cysteine, an alteration that modifies protein thermal stability [105]. In addition, several substitutions in the HIST1H4G histone fold domain enhance the hydrophobic nature of this variant and disrupt several interactions that are essential for nucleosome assembly. For instance, R46H substitution in the histone fold domain was shown to create a less compact nucleosome structure [106]. The C-terminus of HIST1H4G is truncated by five residues that would otherwise stabilize H4 and H2A interactions [107]. In summary, the substitutions in HIST1H4G have been shown to form nucleosomes with an open structure, resulting in less compact chromatin fibers [108,109].

## 5. Dysregulated Histone Genes in Breast Cancer

### 5.1. Histone H2A Variants

Differential gene expression studies reveal that *HIST1H2AC* is upregulated in ER-positive clinical breast cancer tissues and ER-positive cell lines and is associated with poor patient prognosis [110]. This same study showed that *HIST1H2AC* upregulates estrogen receptor target genes *BCL2* and *c-MYC* by recruiting the ER*α* transcription factor to their respective promoter regions. Depleting *HIST1H2AC* impaired the estrogen signaling pathway and reduced cell proliferation [110]. Additionally, breast cancer patients with low *HIST1H2AC* expression benefitted from anthracycline adjuvant chemotherapy, whereas patients with high *HIST1H2AC* expression were resistant to treatment, suggesting that *HIST1H2AC* is linked to anthracycline sensitivity [110].

Other studies have shown that *HIST1H2AH*, *HIST1H2AK*, *HIST1H2AG*, and *HIST1H2AM* are upregulated in breast cancer specimens [111,112,113,114]. Furthermore, in women of Indian descent, the expression levels of *HIST3H2A* relative to normal tissue were higher compared to those of Western patients [115], indicating ethnic diversity in H2A gene expression in breast cancer. Interestingly, *HIST3H2A* expression is considerably higher in the most aggressive breast cancer subtypes, particularly in triple-negative breast cancer and brain metastases of the primary breast cancer [116,117]. In investigating the mechanistic role of elevated histone H2A levels, another study discovered that *HIST2H2AC* was upregulated downstream of the EGFR signaling pathway. This upregulation stimulated oncogenic gene expression in proliferating mammary epithelial (EpH4 and HC11) and breast cancer cells (MC4-L2 and T47-D) [72]. In the same study, silencing of *HIST2H2AC* expression suppressed EGF-induced *Zeb-1* expression and downregulated *E-cadherin*. Thus, aberrant expression of H2A variants can lead to dysregulated expression patterns that promote human diseases such as breast cancer.

### 5.2. Histone H2B Variants

Histone H2B variants are also dysregulated in breast cancer. For instance, *HIST1H2BE* was shown to be upregulated in estrogen-positive breast cancer tumors that exhibited resistance to aromatase inhibitors, indicating a potential association between *HIST1H2BE* and resistance to aromatase inhibitors [118,119]. In separate studies, upregulation of *HIST2H2BC* correlated with paclitaxel resistance in triple-negative breast cancer cells [120], while overexpression of *HIST1H2BK* correlated with increased expression of VEGF165, a vascular endothelial growth factor isoform associated with breast tumor cell invasion of the lungs and bones [121]. Moreover, genome-wide profiling in triple-negative breast cancer patient samples and cell lines revealed overexpression of *HIST1H2BO* in this highly aggressive breast cancer subtype, suggesting its potential role in tumor initiation, maintenance, or progression [113,122]. Bioinformatic studies also associate *HIST1H2BO* overexpression with breast cancer brain and spine metastases, as well as poor overall survival [123,124,125]. *HIST1H2BO* is upregulated in other hormone-dependent cancers and primary brain tumors [126,127,128,129,130,131,132]. In BT-474, MCF7, and ZR-75-1 breast cancer cell lines, histone variants *HIST1H2BF* and *HIST1H2BO* were co-expressed with the phosphatase magnesium-dependent 1 delta protein (PPM1D), a breast cancer protein that correlates with poor prognosis [133]. The *HIST1H2BJ* promoter is hypomethylated in brain metastases of breast cancer, leading to *HIST1H2BJ* over-expression [134]. Differential transcriptome analysis of primary and metastatic tumors revealed *HIST1H2BB*, *HIST1H2BF*, and *HIST1H2BC* as markers associated with breast cancer metastases to the brain and lymph nodes [135,136]. Furthermore, *HIST1H2BB* expression in primary breast cancer tumors was linked with recurrence-free survival [118]. Finally, *HIST1H2BL* was shown to drive histone modification crosstalk to upregulate *c-MYC*, thus promoting tumor cell proliferation [137]. These findings suggest that the expression of several H2B histone variants influences histone PTMs and activates genes and pathways associated with breast cancer.

### 5.3. Histone H3 Variants

In recent years, there has been notable interest surrounding histone H3 variants. Specifically, several studies have shown that H3 variants provide an extra level of control in gene expression by replacing their canonical counterpart, and their dysregulation is highly associated with the acquisition of malignant traits [46,54,138].

Although 10 different H3 genes encode the same H3.1 amino acid sequence, a recent study reported that only the *HIST1H3H* and *HIST1H3D* genes were differentially regulated in metastatic breast cancer [139]. This suggests that at the transcript level, these histone genes may not be functionally redundant [140,141]. *HIST3H3* is also upregulated in breast cancer and is positively associated with activated polymorphonuclear neutrophil (PMN)-induced breast cancer metastasis [142]. Other studies also suggest that several histone variants regulate inflammatory response genes, thus affecting the innate immune response to cancer [143,144]. Indeed, the chronic expression of inflammatory genes can create an environment that is conducive to breast cancer initiation, progression, and invasion [145].

### 5.4. Histone H4 Variants

According to a study of patient samples from The Human Protein Atlas database, breast cancers exhibit considerably higher *HIST1H4G* expression than non-cancerous breast tissue [109]. *HIST1H4G* upregulation was also observed in breast cancer cell lines (MCF7, LCC1, and LCC2) relative to non-cancerous epithelial cells (MCF10A). Relative to other cancers, *HIST1H4G* exhibited the highest expression in breast cancer, suggesting that it is cancer-type-specific. Notably, *HIST1H4G* expression progressively increased as tumors progressed to more advanced stages, even though the expression of canonical H4 did not show significant variation. Furthermore, knocking out *HIST1H4G* reduced tumor formation in a tumor xenograft model [109]. This same study also demonstrated that the nucleolar histone chaperone NPM1 (nucleophosmin/B23) interacts uniquely with HIST1H4G but not with other histone H4 proteins. This suggests that NPM1 is a distinct chaperone for HIST1H4G [109]. The incorporation of HIST1H4G into nucleosome also renders chromatin more accessible and activates global rRNA synthesis [146,147], which is essential for breast cancer cell proliferation.

## 6. Onco-Histones and Breast Cancer

As described above, dysregulated histone variant gene expression is strongly associated with cancers such as breast cancer. Histone genes can also be mutated, with the mutated histones themselves driving oncogenesis, hence the term “onco-histones” [148]. These mutations on histones mechanistically can act by modifying the nucleosome structure, stability, DNA accessibility, and/or histone–protein interactions to cause changes in gene expression and cancer [148]. One of the most common H2A mutations is R29Q [149]. This residue can be di-methylated by the enzyme protein arginine methyltransferase 6 (PRMT6), and di-methylation on the arginine at position 29 (H2A R29me2) has been linked to transcriptional repression as it localizes to transcriptionally inactive chromatin fibers [150]. The adjacent residues K74 and K75 are known to participate in H2A–H3 and H2A–DNA interactions; however, these residues are commonly mutated to K74N and K75N [151,152]. These cancer-associated histone mutations on H2A have been shown to affect nucleosome sliding and enhance remodeling rates [149,153]. Other prevalent onco-histones implicated in breast cancer are H2A E121Q and H2A E121K [154]. E121 is a conserved residue in the H2A C-terminal tail and is involved in the interactions of the H2A–H2B dimer with the H3–H4 tetramer, and with linker DNA [150]. Recently, it was shown that E121 residue forms a salt bridge with H3K14 [155]. Thus, E121 may play a crucial role in maintaining nucleosome stability or mediating histone–protein interactions that regulate chromatin structure and dynamics [156,157]. Mutation of E121 might disrupt key interactions necessary for nucleosome integrity.

H2B isoforms also have relatively high mutation frequencies. One such mutation is E71K, which was shown to inhibit the differentiation of mesenchymal progenitor cells [149] and is most often associated with breast cancer [154,158], where it activates *ADAM19* and genes critical for cancer invasion [159]. In vitro, the H2BE71K onco-histone can combine with H2A to form a dimer. However, the process of nucleosome assembly was hindered as the H2A–H2B E71K dimer was unable to create stable histone octamers with H3 and H4 [149,154]. Other hotspot mutations on H2B include E76K and E76Q, which mechanistically weaken H2B–H4 interactions and are also observed in breast cancer [154]. These E76K/Q mutations occur most frequently in *HIST1H2BC*, *HIST1H2BD*, and *HIST1H2BH* variants and not so frequent in *HIST1H2BB*, *HIST1H2BJ*, *HIST1H2BK,* and *HIST1H2BO* [158]. The expression of the E76K onco-histone in a non-cancerous breast epithelial cell line (MCF10A) altered chromatin accessibility at gene regulatory elements, leading to enhanced colony formation (an oncogenic phenotype) [154]. Other studies show that H2B E2 is a site of ADP-ribosylation, and that this post-translational modification is associated with increased chromatin relaxation with potential implication in DNA repair and gene regulation [160,161]. Interestingly, E2 is frequently mutated in cancers [149,154]; nevertheless, the malignant potential of this oncogenic mutation is yet to be investigated [154,162,163]. Additionally, the E113K/Q mutation occurs frequently in breast cancer [154,162,163], though its mechanistic role is still unclear. One might hypothesize that because this mutation occurs in the acidic patch of H2B, a region in histones known as the ‘landing dock’ for chromatin remodelers [164], it might affect their binding and thereby affect the balance between active and repressive chromatin states. Another recent study shows that the expression of the onco-histone H2B D51A/N significantly enhanced cell proliferation in breast cancer [165]. H2B D51 is an ADP-ribosylation site that is essential for p300-mediated acetylation inhibition of several lysine residues on H2B. Changes to acetylation patterns of histone H2B and histone acetyltransferases activity is common in breast cancer [166,167]. Therefore, the loss of ADP-ribosylation on H2B D51A/N mutations might disrupt the acetylation pattern on H2B, leading to significant changes in chromatin accessibility at enhancers and promoters, along with alterations in gene expression patterns. Notably, mutation of D51 to A was associated with accelerated breast tumor formation in mouse xenografts [165]. These discoveries imply that both the presence and the specific mutations of these variants play crucial roles in organizing chromatin structure, which in turn influences patterns of gene expression.

Mutations on H3 were among the first mutations discovered in histones and are associated with cancers. H3 mutations such as K27M, K36M, and G34V/R have been discovered by many groups [33,168,169,170,171]. H3 K27M is linked to many cancers including pediatric glioblastoma (pGBM) [172], adult diffuse midline glioma [170], head and neck squamous cell carcinoma [173], melanoma [174], and acute myeloid leukemia [175]. H3 K27M can directly modulate nucleosome assembly but also indirectly modulate the methylation status of histone lysine residues [169,176,177]. In detail, the K27M mutants alter normal H3 methylation patterns, disrupting PRC2-mediated repressive function and the enhancer landscape, leading to widespread epigenetic dysregulation and tumorigenic gene expression profiles [168,178,179,180]. In general, while the N-terminal tail mutations such as K27M, K36M, and G34 have been extensively examined across numerous malignancies, their frequencies and implications, if any, in breast cancer are yet to be investigated. Although the functional implications of mutations linked with the replication-independent histone variant H3.3 have been widely researched in several tumor types and have attracted significant interest, research on mutations in other H3 variants such as H3.2 and HIST3H3 is still in its early stages [171].

Histone H4 genes on the other hand are rarely mutated in cancers. The most common H4 onco-histone carries an R4C mutation, with equal prevalence across all H4 genes [33,149]. R4C is also a site of symmetric demethylation and citrullination, which serve as marks for transcriptional repression and DNA damage. Disrupting these PTMs is associated with oncogenesis [181,182,183]. H4 D67H/N and R93T mutations are also found in cancer patients [148]. These residues form a hydrogen bond with H2B to stabilize the nucleosome structure [184], so altering these residues may result in nucleosome instability.

Overall, onco-histone mutations not only alter chromatin structure but also influence potential post-translational modifications (PTMs) and interactomes, leading to changes in gene expression. Recent studies have begun to elucidate how these onco-histones impact chromatin structure and remodeling. For instance, a dimer exchange assay facilitated by the nucleosome assembly protein (Nap1) demonstrated that histone mutations at the dimer–tetramer interface between histones H2B and H4 were destabilizing. These destabilizing mutations include E56 and R29 on histone H2A; D68, E71, and E76 on histone H2B; and E50 and E9 on histone H3. Additionally, some onco-histones inhibit the formation of stable octamers, such as H2BD68A/N, H2BE76K, H3E50K, and H3E97A/K [148,149,157,185]. These effects in vivo ultimately impact chromatin structure, resulting in significant consequences for gene expression.

## 7. Epigenetic Inhibitors and Clinical Trials

The incorporation of histone variants into chromatin can induce local and global changes in PTMs, ultimately affecting the histone interaction network as discussed in the previous sections. Therapeutic avenues that target several histone modifiers to alter acetylation and methylation pattern at anti-tumor gene promoters are being actively explored across different tumors. Several of these compounds have shown remarkable efficacy in treating breast cancer, both alone and in combination with other chemotherapeutic drugs and immunotherapy. We summarize some of these therapies below.

(a) DNA methyltransferase inhibitors (DNMTis): DNA methyltransferases (DNMTs) are enzymes that add a methyl group to the fifth carbon of cytosine residues in DNA for gene regulation. Aberrant DNA methylation patterns are linked to various diseases, including cancer. Targeting these patterns could help re-establish normal methylation and correct dysregulation. Currently, two types of DNMTis are used: 1. DNMTis that inhibit the enzymatic function of DNMTs and 2. cytosine analogs that incorporate into DNA and replace the carbon at position 5 of cytosine (C-5) with N-5, disabling DNMTs. These compounds reverse the DNA hypermethylation status of tumor suppressor gene promoters and reactivate their expression in cancerous cells [186,187]. These epi-drugs have been the focus of extensive research, including 5-azacytidine, SGI-110, hydralazine, the antisense oligonucleotide MG98, decitabine, and zebularine [188,189,190,191,192,193,194].

(b) Histone deacetylase inhibitors (HDACis) are a class of epigenetic anticancer therapeutics that target histone deacetylase enzymes. Deacetylation of histones at tumor suppressor genes can render the chromatin more compact and transcriptionally inactive. HDAC inhibitors can rectify the aberrant acetylation status of histones in cancers leading to reactivation of anti-oncogenes. Various cancer types, including breast cancers, have shown a positive response to HDACis because of their effectiveness in inhibiting tumor growth by increasing cell apoptosis. Several of these epigenetic compounds have shown promising outcomes for advanced breast cancer treatment during clinical trials, including entinostat, abexinostat, givinostat, and vorinostat [195,196,197,198,199,200].

(c) Histone methyltransferases inhibitors (HMTis) target histone lysine methyltransferases (KMTs) and arginine methyltransferases (PRMTs), which are enzymes that add methyl groups to lysine and arginine residues on histones. Various HMT inhibitors have shown significant anti-tumor effects in clinical studies. A significant number of these inhibitors target the function of EZH2, the catalytic subunit of polycomb repressive complex 2 (PRC2), which silences target gene expression by methylating histone 3 at lysine 27 (H3K27me3) in multiple cancers [201,202]. Some of these epi-enzyme inhibitors, such as pinometostat and tazemetostat, have been evaluated in clinical trials and showed promising results in breast tumors [203,204,205]. 

(d) Bromodomain and extra-terminal inhibitors (BETis) target a family of epigenetic readers that consist of two N-terminal tandem bromodomains and a C-terminal extra-terminal motif [206]. This family consists of bromodomain-containing proteins (BRD) such as BRD2, BRD3, BRD4, and the bromodomain testis associated protein (BRDT), which can form a complex with HDACs to control transcription through processes such as histone acetylation, chromatin remodeling, and recruitment of other transcriptional machinery [207,208,209]. One of the BETs that is garnering the most attention is BRD4, which is known to promote the transcriptional initiation and elongation by binding to hyper-acetylated promoters and super-enhancers to activate several oncogenes [210,211]. Several effective inhibitors that interfere with the binding of BETs to acetylated histones have shown promising outcomes, including but not limited to JQ1, I-BET762, TEN-010, and OTX-015 [212,213,214,215].

## 8. Conclusions and Future Perspective

This review focuses on histone variants implicated in breast cancer. Histone variants exhibit tissue- and time-dependent expression patterns and are incorporated into chromatin with the assistance of chaperones, often replacing canonical histones during or after cell division. Recent studies highlight significant dysregulation of histone variants in breast cancer and other diseases, underscoring their diverse cellular functions. Moreover, increasing evidence suggests varying mutation frequencies among histone variants within the same gene family. Surprisingly, even a single amino acid substitution in histone variants (including onco-histones) can markedly disrupt nucleosome stability, chromatin structure, DNA–nucleosome interactions, and DNA accessibility. Additionally, histone variants may carry distinct post-translational modifications that contribute to dysregulated chromatin remodeling networks and gene expression, thereby promoting tumorigenesis.

Fortunately, unlike genetic mutations, epigenetic aberrations have the potential to be reversible, providing new therapeutic avenues for cancer cell management. Indeed, the use of epigenetic targets in combination with conventional chemotherapeutic drugs is emerging as an effective technique for increasing anticancer activity, reducing drug resistance, and bolstering the host immune response [216,217,218,219]. Thus, we propose a deeper exploration of histone variants, their influence on nucleosome structure, and the downstream pathways involved in dysregulating cancer-related genes, especially in cases lacking evident DNA mutations in well-established breast cancer oncogenes.

## Data Availability

Data sharing is not applicable to this article as no new data were created or analyzed in this study.

## Appendix A

**Table A1:** Histone variant gene and protein names and their chromosomal locations.

Chromosome	Name	Gene Name	Protein Name
1q21.2	H2A clustered histone 18	*H2AC18*	HIST2H2AA
1q21.2	H2A clustered histone 19	*H2AC19*	HIST2H2AA4
1q21.2	H2A clustered histone 20	*H2AC20*	HIST2H2AC
1q21.2	H2A clustered histone 21	*H2AC21*	HIST2H2AB
1q42.13	H2A clustered histone 25	*H2AC25*	HIST3H2A
1q21.2	H2B clustered histone 18	*H2BC18*	HIST2H2BF
1q21.2	H2B clustered histone 19, pseudogene	*H2BC19P*	HIST2H2BD
1q21.2	H2B clustered histone 20, pseudogene	*H2BC20P*	HIST2H2BC
1q21.2	H2B clustered histone 21	*H2BC21*	HIST2H2BE
1q42.13	H2B clustered histone 26	*H2BC26*	HIST3H2BB
1q42.13	H2B clustered histone 27, pseudogene	*H2BC27P*	HIST3H2BA
1q21.1	H3.7 histone	*H3-7*	HIST2H3PS2
1q21.2	H3 clustered histone 13	*H3C13*	HIST2H3D
1q21.2	H3 clustered histone 14	*H3C14*	HIST2H3C
1q21.2	H3 histone clustered 15	*H3C15*	HIST2H3A
1q42.12	H3.3 histone A	*H3-3A*	H3F3A
1q42.13	H3.4 histone, cluster member	*H3-4*	HIST3H3
1q21.2	H4 clustered histone 14	*H4C14*	HIST2H4A
1q21.2	H4 clustered histone 15	*H4C15*	HIST2H4B
2p23.3	Centromere protein A	*CENPA*	CENP-A
3q21.3	H1.10 linker histone	*H1-10*	H1FX
3q22.1	H1.8 linker histone	*H1-8*	H1FOO
4q23	H2A.Z variant histone 1	*H2AZ1*	H2AFZ
5q31.1	macroH2A.1 histone	*MACROH2A1*	H2AFY
5q13.2	H2B.L histone variant 1, pseudogene	*H2BL1P*	H2BP4
5p15.1	H3.Y histone 1	*H3Y1*	H3.Y
5p15.1	H3.Y histone 2	*H3Y2*	H3.X
6p22.2	H1.1 linker histone, cluster member	*H1-1*	HIST1H1A
6p22.2	H1.2 linker histone, cluster member	*H1-2*	HIST1H1C
6p22.2	H1.3 linker histone, cluster member	*H1-3*	HIST1H1D
6p22.2	H1.4 linker histone, cluster member	*H1-4*	HIST1H1E
6p22.1	H1.5 linker histone, cluster member	*H1-5*	HIST1H1B
6p22.2	H1.6 linker histone, cluster member	*H1-6*	HIST1H1T
6p22.2	H1.12 linker histone, cluster member, pseudogene	*H1-12P*	HIST1H1PS1
6p22.2	H2A clustered histone 1	*H2AC1*	HISTH2AA
6p22.2	H2A clustered histone 2, pseudogene	*H2AC2P*	HIST1H2APS1
6p22.2	H2A clustered histone 3, pseudogene	*H2AC3P*	HIST1H2APS2
6p22.2	H2A clustered histone 4	*H2AC4*	HIST1H2AB
6p22.2	H2A clustered histone 5, pseudogene	*H2AC5P*	HIST1H2APS5
6p22.2	H2A clustered histone 6	*H2AC6*	HIST1H2AC
6p22.2	H2A clustered histone 7	*H2AC7*	HIST1H2AD
6p22.2	H2A clustered histone 8	*H2AC8*	HIST1H2AE
6p22.2	H2A clustered histone 9, pseudogene	*H2AC9P*	HIST1H2APS3
6p22.2	H2A clustered histone 10, pseudogene	*H2AC10P*	HIST1H2APS4
6p22.1	H2A clustered histone 11	*H2AC11*	HIST1H2AG
6p22.1	H2A clustered histone 12	*H2AC12*	HIST1H2AH
6p22.1	H2A clustered histone 13	*H2AC13*	HIST1H2AI
6p22.1	H2A clustered histone 14	*H2AC14*	HIST1H2AJ
6p22.1	H2A clustered histone 15	*H2AC15*	HIST1H2AK
6p22.1	H2A clustered histone 16	*H2AC16*	HIST1H2AL
6p22.1	H2A clustered histone 17	*H2AC17*	HIST1H2AM
6p22.2	H2B clustered histone 1	*H2BC1*	HIST1H2BA
6p22.2	H2B clustered histone 2, pseudogene	*H2BC2P*	HIST1H2BPS1
6p22.2	H2B clustered histone 3	*H2BC3*	HIST1H2BB
6p22.2	H2B clustered histone 4	*H2BC4*	HIST1H2BC
6p22.2	H2B clustered histone 5	*H2BC5*	HIST1H2BD
6p22.2	H2B clustered histone 6	*H2BC6*	HIST1H2BE
6p22.2	H2B clustered histone 7	*H2BC7*	HIST1H2BF
6p22.2	H2B clustered histone 8	*H2BC8*	HIST1H2BG
6p22.2	H2B clustered histone 9	*H2BC9*	HIST1H2BH
6p22.2	H2B clustered histone 10	*H2BC10*	HIST1H2BI
6p22.1	H2B clustered histone 11	*H2BC11*	HIST1H2BJ
6p22.1	H2B clustered histone 12	*H2BC12*	HIST1H2BK
6p22.1	H2B clustered histone 13	*H2BC13*	HIST1H2BL
6p22.1	H2B clustered histone 14	*H2BC14*	HIST1H2BM
6p22.1	H2B clustered histone 15	*H2BC15*	HIST1H2BN
6p22.1	H2B clustered histone 16, pseudogene	*H2BC16P*	HIST1H2BPS2
6p22.1	H2B clustered histone 17	*H2BC17*	HIST1H2BO
6p22.2	H3 clustered histone 1	*H3C1*	HIST1H3A
6p22.2	H3 clustered histone 2	*H3C2*	HIST1H3B
6p22.2	H3 clustered histone 3	*H3C3*	HIST1H3C
6p22.2	H3 clustered histone 4	*H3C4*	HIST1H3D
6p22.2	H3 clustered histone 5, pseudogene	*H3C5P*	
6p22.2	H3 clustered histone 6	*H3C6*	HIST1H3E
6p22.2	H3 clustered histone 7	*H3C7*	HIST1H3F
6p22.2	H3 clustered histone 8	*H3C8*	HIST1H3G
6p22.2	H3 clustered histone 9, pseudogene	*H3C9P*	HIST1H3PS1
6p22.1	H3 clustered histone 10	*H3C10*	HIST1H3H
6p22.1	H3 clustered histone 11	*H3C11*	HIST1H3I
6p22.1	H3 clustered histone 12	*H3C12*	HIST1H3J
6p22.2	H4 clustered histone 1	*H4C1*	HIST1H4A
6p22.2	H3 clustered histone 2	*H4C2*	HIST1H4B
6p22.2	H3 clustered histone 3	*H4C3*	HIST1H4C
6p22.2	H3 clustered histone 4	*H4C4*	HIST1H4D
6p22.2	H3 clustered histone 5	*H4C5*	HIST1H4E
6p22.2	H3 clustered histone 6	*H4C6*	HIST1H4F
6p22.2	H3 clustered histone 7	*H4C7*	HIST1H4G
6p22.2	H3 clustered histone 8	*H4C8*	HIST1H4H
6p22.1	H3 clustered histone 9	*H4C9*	HIST1H4I
6p22.1	H3 clustered histone 10, pseudogene	*H4C10P*	HIST1H4PS1
6p22.1	H3 clustered histone 11	*H4C11*	HIST1H4J
6p22.1	H3 clustered histone 12	*H4C12*	HIST1H4K
6p22.1	H3 clustered histone 13	*H4C13*	HIST1H4L
7p13	H2A.Z variant histone 2	*H2AZ2*	H2AFV
7q36.1	H2B.K variant histone 1	*H2BK1*	H2BE1
10q22.1	macroH2A.2 histone	*MACROH2A2*	H2AFY2
11q23.3	H2A.X variant histone	*H2AX*	H2AFX
12q13.11	H1.7 linker histone	*H1-7*	H1FNT
12p12.3	H2A.J histone	*H2AJ*	H2AFJ
12p11.21	H3.5 histone	*H3-5*	H3F3C
12p12.3	H4 histone 16	*H4C16*	HIST4H4
17q21.33	H1.9 linker histone, pseudogene	*H1-9P*	HILS1
17q11.2	H2B.N variant histone	*H2BN1*	H2B.N
17q25.1	H3.3 histone B	*H3-3B*	H3F3B
21q22.3	H2B clustered histone 12 like	*H2BC12L*	H2BFS
22q13.1	H1.0 linker histone	*H1-0*	H1FV
Xq28	H2A.B variant histone 1	*H2AB1*	H2AFB1
Xq28	H2A.B variant histone 2	*H2AB2*	H2AFB2
Xq28	H2A.B variant histone 3	*H2AB3*	H2AFB3
Xp21.1	H2A.L variant histone 1M, pseudogene	*H2AL1MP*	H2AL1SP
Xp21.1	H2A.L variant histone 1Q	*H2AL1Q*	H2AL1QP
Xp11.4	H2A.L variant histone 3	*H2AL3*	H2AL1RP
Xp11.4	H2A.P histone	*H2AP*	HYPM (CXorf27)
Xq26.3	H2A.Q variant histone 1, pseudogene	*H2AQ1P*	
Xq22.2	H2B.W histone 1	*H2BW1*	H2BFWT
Xq22.2	H2B.W histone 2	*H2BW2*	H2BFM
Xq22.2	H2B.W histone 3, pseudogene	*H2BW3P*	
Xq22.2	H2B.W histone 4, pseudogene	*H2BW4P*	H2BFXP
